# Development and Validation of an Electronic Frailty Index Using Routine Electronic Health Records: An Observational Study From a General Hospital in China

**DOI:** 10.3389/fmed.2021.731445

**Published:** 2021-09-28

**Authors:** Yao-Dan Liang, Yi-Bo Xie, Ming-Hui Du, Jing Shi, Jie-Fu Yang, Hua Wang

**Affiliations:** ^1^Department of Cardiology, Beijing Hospital, National Center of Gerontology, Institute of Geriatric Medicine, Chinese Academy of Medical Sciences, Beijing, China; ^2^Department of Pulmonary and Critical Care Medicine, Beijing Hospital, National Center of Gerontology, Institute of Geriatric Medicine, Chinese Academy of Medical Sciences, Beijing, China; ^3^Information Center, Beijing Hospital, National Center of Gerontology, Institute of Geriatric Medicine, Chinese Academy of Medical Sciences, Beijing, China; ^4^Beijing Institute of Geriatrics, Beijing Hospital, National Center of Gerontology, Institute of Geriatric Medicine, Chinese Academy of Medical Sciences, Beijing, China

**Keywords:** frailty, electronic health records-EHR, long hospital stay, mortality, hospitalized costs

## Abstract

**Background:** This study aimed to develop and validate an electronic frailty index (eFI) based on routine electronic health records (EHR) for older adult inpatients and to analyze the correlations between frailty and hospitalized events and costs.

**Methods:** We created an eFI from routine EHR and validated the effectiveness by the consistency of the comprehensive geriatric assessment-frailty index (CGA-FI) with an independent prospective cohort. Then, we analyzed the correlations between frailty and hospitalized events and costs by regressions.

**Results:** During the study period, 49,226 inpatients were included in the analysis, 42,821 (87.0%) of which had enough data to calculate an eFI. A strong correlation between the CGA-FI and eFI was shown in the validation cohort of 685 subjects (Pearson's *r* = 0.716, *P* < 0.001). The sensitivity and specificity for an eFI≥0.15, the upper tertile, to identify frailty, defined as a CGA-FI≥0.25, were 64.8 and 88.7%, respectively. After adjusting for age, sex, and operation, an eFI≥0.15 showed an independent association with long hospital stay (odds ratio [OR] = 2.889, *P* < 0.001) and death in hospital (OR = 19.97, *P* < 0.001). Moreover, eFI values (per 0.1) were positively associated with total costs (β = 0.453, *P* < 0.001), examination costs (β = 0.269, *P* < 0.001), treatment costs (β = 0.414, *P* < 0.001), nursing costs (β = 0.381, *P* < 0.001), pharmacy costs (β = 0.524, *P* < 0.001), and material costs (β = 0.578, *P* < 0.001) after adjusting aforementioned factors.

**Conclusions:** We successfully developed an effective eFI from routine EHR from a general hospital in China. Frailty is an independent risk factor for long hospital stay and death in hospital. As the degree of frailty increases, the hospitalized costs increase accordingly.

## Introduction

As the population of older adults rises globally, the condition of frailty is gaining prominent attention (1). In China, the older adults aged 65 years and older has reached 190 million, accounting for 13.5% of the total population at the end of 2020 (2). Frailty describes a decline in physiological capacity across multiple organ systems, characterized by an increased vulnerability to stressors (3). The condition of frailty is associated not only with a myriad of adverse outcomes (4) but also with increased healthcare costs (5–16). However, there is no gold standard assessment instrument for frailty (17, 18). The most widely used instruments in clinical research are variations of the frailty phenotype (19) or frailty index (FI) based on the cumulative deficit model (20). Our previous study showed that the comprehensive geriatric assessment-FI (CGA-FI) may be an optimal assessment tool among five prevalent frailty measurements (21). Although numerous clinicians have embraced the concept of frailty, we are facing large challenges to make the translation from frailty research to clinical practice. The barriers include not only a lack of consensus among prevalent frailty measurements but also time and resource limitations in busy clinical environments.

To reduce the burden of frailty assessment on clinicians, Clegg et al. developed an electronic FI (eFI) using routine electronic health records (EHR) in UK primary care (22). Based on Clegg's eFI, Pajewski and his colleagues modeled another adapted eFI with more information on nursing and laboratory assessments using routine EHR in the US, which showed an independent predictive value for mortality, hospitalizations, emergency department visits, and injurious falls (23). Meanwhile, these time-saving assessments of frailty based on EHR have shown good consistency with traditional assessment methods, such as the Fried phenotype and CGA-FI (24, 25). However, there has been no relevant frailty research based on EHR in China, so we do not know whether an effective eFI can be developed from routine EHR in China.

Previous studies on frailty in inpatients have mainly focused on the population in a certain department or with a particular disease type, which cannot fully reflect conditions across all departments. Our previous research has found that the prevalence of frailty between medical and surgical departments was significantly different (21), but there has been no research that can show in detail the distribution of frailty in each department of a general hospital. Moreover, due to limitations in data acquisition, there is currently no study using EHR to analyze the relationship between frailty and hospitalized costs. Therefore, the effective utilization of EHR big data is particularly important, which can provide valuable information for clinical management.

This study aimed to develop and validate an eFI based on routine EHR for older adult inpatients and to analyze the correlations between frailty and hospitalized events and costs.

## Materials and Methods

### Study Design

We conducted a cohort study on frailty using 6-year EHR of older adult inpatients from a general hospital in Beijing, China. The hospital is public, owned by government, with great attention to geriatrics and gerontology. We used a two-step approach to develop and validate an eFI based on the routine EHR for older adult inpatients. First, we followed published guidance on creating a FI using the cumulative deficit model to create an eFI from routine EHR, which involved diagnosis, nursing assessment, and laboratory tests (26). Second, in a validation cohort, we tested whether the eFI identified similar people as the CGA-FI. Then, we analyzed the correlation between frailty and hospitalized events and costs using the routine EHR.

### Population in the EHR

**Figure 1:**
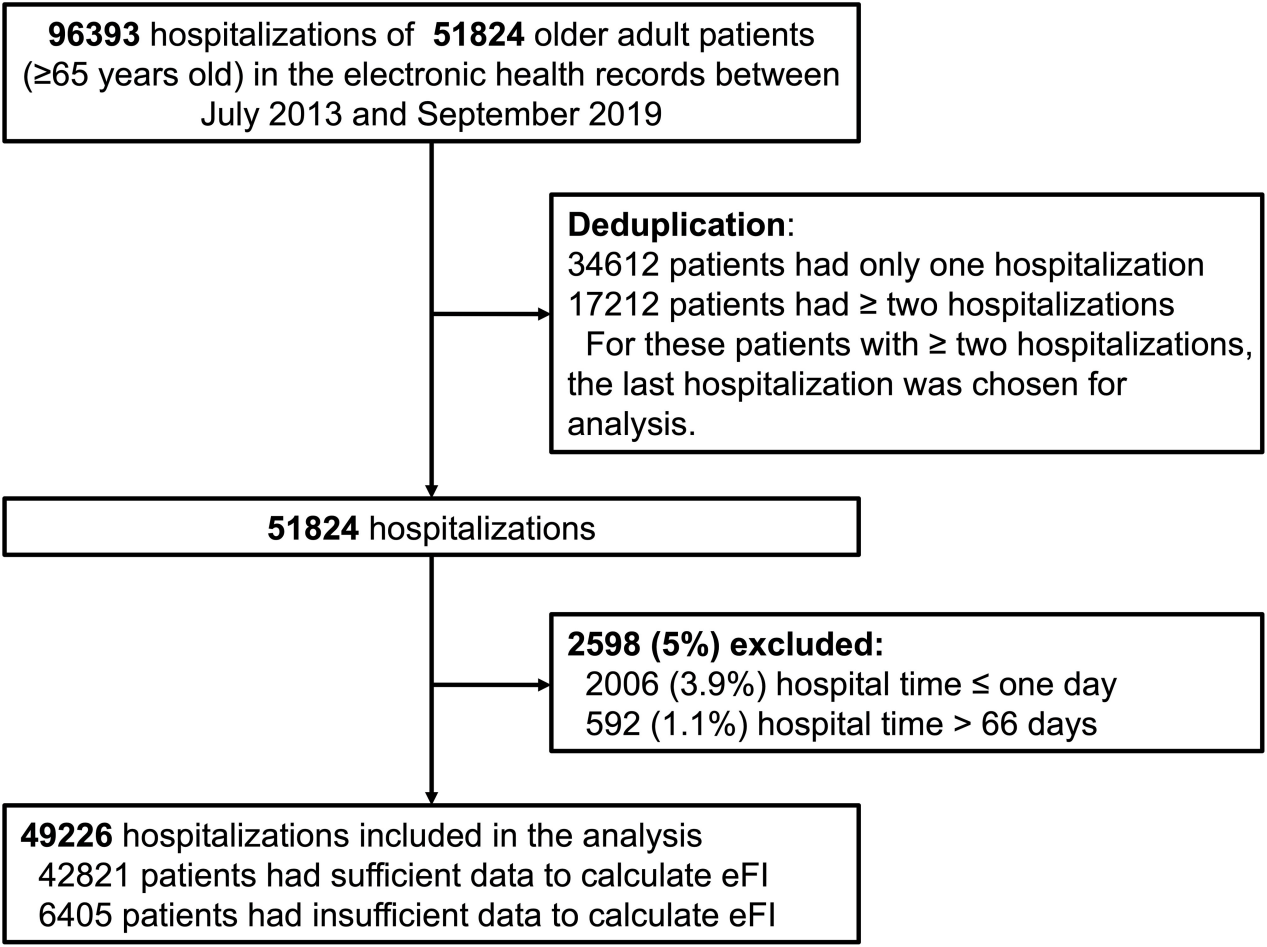
Flow chart of the study cohort.

The patients included were at least 65 years of age in the routine EHR database from July 1, 2013 to September 30, 2019. There were 96,393 hospitalizations involving 51,824 older adult inpatients in the records. During the study period, 34,612 patients had only one hospitalization, while 17,212 patients had two or more hospitalizations in the records. To avoid duplication, we chose the last hospitalization for these patients with two or more hospitalizations in the records for analysis. Considering the meaninglessness of hospital stays that were extremely short (1 day or less) and special issues regarding hospital stays that were extremely long, we excluded 5% of the patients, which included 2,006 (3.9%, 2006/51824) patients with hospital stay ≤ one day and 592 (1.1%, 592/51824) patients with hospital stay > 66 days. Therefore, 49,226 inpatients were eligible (Figure 1).

### Validation Cohort

For the validation exercise, we used a linked dataset on a prospective cohort of inpatients who had been assessed with the research standard CGA-FI. These participants were enrolled from September 2018 to February 2019 in the same hospital. The characteristics of the prospective cohort were described in detail in our previously published article (21). Among the 1,000 older adult patients in the cohort study, 703 participants were matched in the EHR database of 49,226 cases by hospitalized ID and admission time. There were 18 participants who did not have sufficient data to calculate the eFI. Finally, there were 685 older adult inpatients who had both the CGA-FI and eFI assessed. The CGA-FI was the result of the sum of all 48 variables' scores divided by 48. The threshold of 0.25 as the cut-off value of CGA-FI for identifying frailty. The details of all variables in CGA-FI are described in Supplementary Table 1.

### Composition of the Electronic Frailty Index (eFI)

As shown in Supplementary Table 2, according to the core criteria of the Rockwood frailty index (26), we modeled our adapted eFI referring to the factors included in the eFI of Pajewski et al. (23). Forty-five variables were selected to construct the eFI, which included 20 items on the diagnosis of chronic diseases, 20 items on nursing assessments, and 5 items on laboratory tests.

These 20 items on the diagnosis of chronic diseases covered a range of systems, included hypertension, heart failure, myocardial infarction, atrial fibrillation/atrial flutter, peripheral arterial disease, venous thromboembolism, chronic lung disease, peptic ulcer, chronic kidney disease, diabetes, thyroid dysfunction, stroke, Parkinson's disease/parkinsonism, dementia, anxiety, depression, osteoporosis, arthritis, spondylosis/disc disorders, and malignancy. The international Classification of Diseases-tenth version (ICD-10) codes for each diagnosis were determined by three clinicians after discussion and according to the global standard for diagnostic health information from the World Health Organization website (https://icd.who.int/browse10/2010/en#/) (Supplementary Table 3).

These 20 items from the nursing assessments on the day of admission included the Barthel Index (containing 10 items: feeding, bathing, grooming, dressing, bowels, bladder, toilet use, transfers, mobility) (27), visual impairment, hearing impairment, insomnia, consciousness statement, constipation, appetite, pressure ulcer, BMI, heart rate, and blood pressure. The detailed cut-off values are shown in Supplementary Table 2.

Given the importance of laboratory tests for frailty assessment (28, 29), we included 5 routine laboratory items for constructing the eFI. Based on our previous study (21), we found that hemoglobin, albumin, sodium, and D-dimer were independently associated with frailty. Meanwhile, qualitative analysis of urine protein was widely tested and was useful to reflect renal function. Therefore, we selected these abovementioned 5 items from the routine laboratory tests. Usually, the blood and urine specimens were tested within 24 h after admission for inpatients. If a patient had more than one result for the same item, we chose the first result to construct the eFI. The detailed cut-off values are shown in Supplementary Table 2.

The eFI included a total of 45 factors and was calculated as the unweighted sum of the score for each factor, divided by the total number of non-missing items. There were no missing values regarding the diagnosis for all inpatients. Moreover, we did not want an absence of the Barthel Index, which contained 10 items and necessarily implied functional status. Therefore, these abovementioned 30 items in the eFI were guaranteed. We additionally excluded individuals who did not have at least 6 of the 10 items on the nursing assessments (except Barthel Index) or at least 3 of the 5 items on the laboratory measurements. Finally, we required ≥39 non-missing items, which were consistent with recommendations for constructing the FI (26). The missing data elements from the routine EHR in calculating the eFI are described in detail in Supplementary Table 4. The upper tertile of the eFI scores (eFI ≥ 0.15) was defined as the cut-off value to indicate frailty.

### Hospitalized Events and Costs

We defined hospitalized events as long hospital stay (>14 days) and death in hospital. Hospitalized costs included total costs, examination costs, treatment costs, nursing costs, pharmacy costs, and material costs. The total cost was the sum of all payments to the hospital for this hospitalization. Examination costs meant the payment for all examinations, such as laboratory measurements and imaging examinations. Treatment costs referred to payments for treatments, mainly by doctors and therapists, such as operation fees and consultation fees. Nursing costs referred to payments for all nursing care. Pharmacy costs referred to payments for all medications used during this hospitalization. Material costs referred to the payment for all medical consumable materials, of which surgical and interventional consumables accounted for a large proportion. We calculated hospitalized costs in United States dollars using the average exchange rate from 2013 to 2019 (1 USD = 6.46 CNY) for international comparisons.

### Statistical Analysis

Descriptive statistics were compared between the groups using chi-square tests for categorical variables and *t*-tests or Mann-Whitney *U* for continuous variables. Pearson's correlation coefficient was used to describe the association between the continuous versions of the eFI and the CGA-FI. Additionally, a receiver operating characteristic (ROC) curve was calculated to estimate the area under the curve (AUC) for the eFI in relation to frailty, which was defined as CGA-FI≥0.25. We examined the association between frailty (eFI≥0.15) and hospitalized events using multivariate logistic regression, adjusting for age, sex, and operation [any surgery or intervention treatment in this hospitalization). Considering the influence of departments on hospitalization events, we divided 30 clinical departments into five groups (1) cardiology department; (2) internal medicine departments except cardiology; (3) orthopedics department; (4) surgical departments except orthopedics; (5) emergency department and intensive care units (ICUs)] and assessed the prevalence of frailty in different departments. We conducted a separate analysis for each subgroup. The results of the logistic regression models are presented as odds ratios (ORs) and 95% confidence intervals (95% CIs).

To investigate the impact of the eFI on hospitalized costs, generalized linear regression models (gamma-distributed and log-linked) adjusted for age, sex, and operation were used for all patients and each subgroup. For patients with two or more hospitalizations in the records, we chose the last hospitalization for analysis. The eFI values were multiplied by 10 to give equal 0.1 increments in the generalized linear regression models. The use of a gamma-distributed generalized linear model with a log-transformed link function has been shown to be a good method to estimate health-care cost distributions that are generally right-skewed (30). The results of the generalized linear regression models were presented as the β and 95% CIs for eFI values (per 0.1). A *P* < 0.05 was considered statistically significant.

We performed sensitivity analyses to assess the robustness of the eFI as an independent associated factor of hospitalized events and costs. For patients with two or more hospitalizations (17,212 out of 51,824 patients, 33.2%), we used the first hospitalization records instead of the last hospitalization records. We also excluded 5% patients with extremely short hospital stay (3.2%, 1,0656/51,824) or extremely long hospital stay (1.8%, 958/51,824). According to the method of constructing the eFI, 87.6% (43,116/49,210) of patients had sufficient data to calculate the eFI. For comparison with the original results, we still used 0.15 as the cut-off point of the eFI to identify frailty. Other statistical methods in the sensitivity analysis were the same as in the original analysis.

All analyzes were performed using the R software program, version 3.5.3.

## Results

### Demographic and Clinical Characteristics

**Figure 2:**
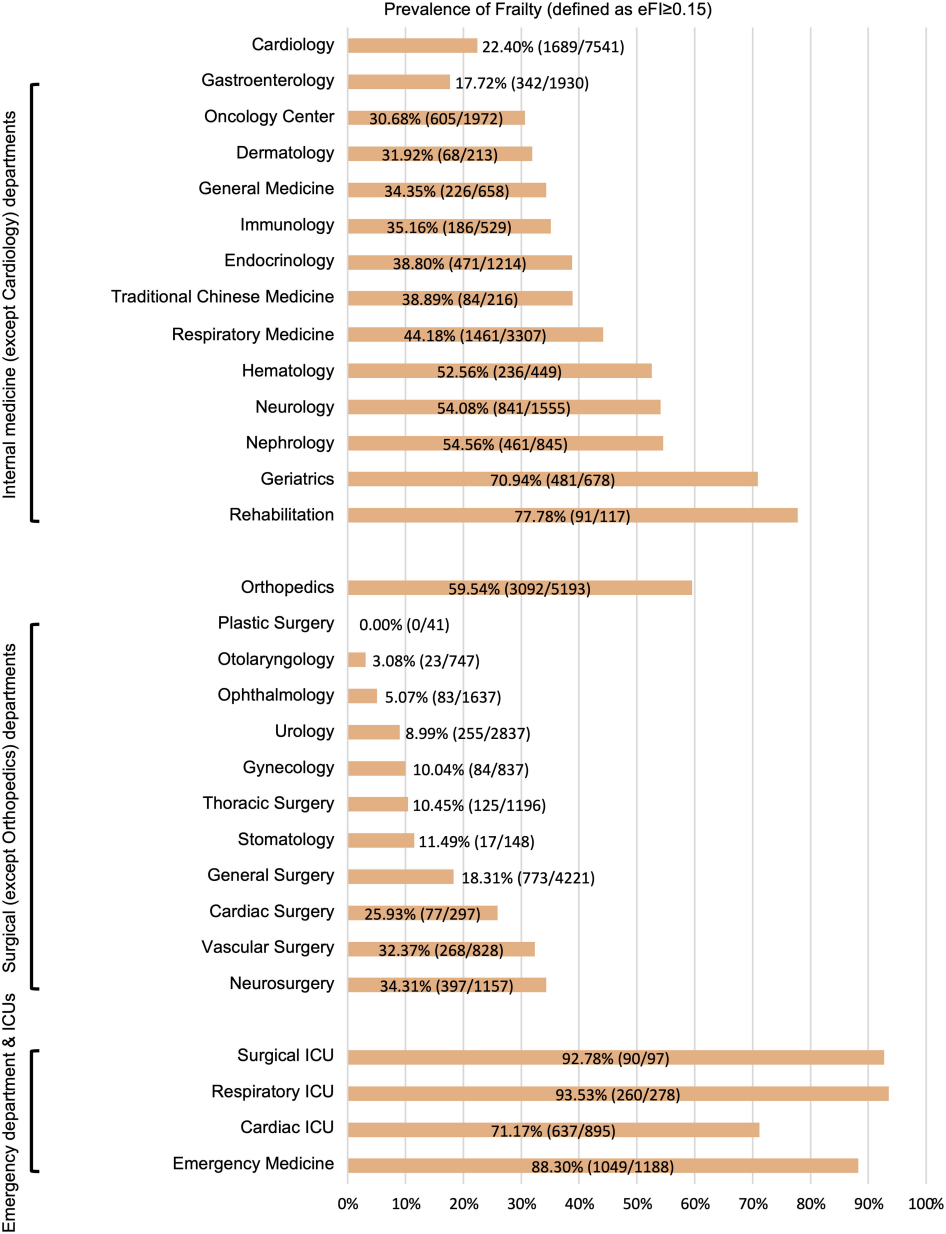
Prevalence of frailty in different departments. eFI, electronic frailty index; ICUs, intensive care units.

During the study period, 49,226 older adult inpatients from the EHR database were included (Figure 1). There were 50.5% males and the average age was 74.8 ± 6.9 years old. According to the method of constructing the eFI, there were 42,821 (87.0%) patients with sufficient data to calculate the eFI and 6,405 (13.0%) patients with insufficient data. The two groups had similar ages (74.8 ± 7.0 vs. 75.1 ± 6.7 years) and proportions of males (50.5 vs. 50.7%). However, the hospital days (9 [6, 14] vs. 4 [3, 7] days; *P* < 0.001) and death in hospital (3.4 vs. 0.7%; *P* < 0.001) were significantly different in these two groups. Moreover, the hospitalized costs in patients with insufficient data were significantly lower than those in the other group (all *P* < 0.01). Detailed information on the entire population is shown in Table 1. The departments of ophthalmology (54.5%, 3491/6405), general surgery (20.0%, 1284/6405), and traditional Chinese medicine (7.9%, 506/6405) were the top three leading contributors to the medical records with insufficient data (Supplementary Table 5). Among the 42,821 patients, 33.8% had an eFI≥0.15, and the median eFI was 0.111 (Table 1). Figure 2 shows the prevalence of frailty in different departments. Females showed a significantly higher proportion of frailty than males (36.4 vs. 31.2%; *P* < 0.001).

**Table 1:** Demographics and hospitalized events and costs of all patients stratified by whether or not the eFI could be calculated from the electronic health records.

	**All patients**	**Sufficient data to calculate eFI**	**Insufficient data to calculate eFI**	***P* values**
	***n* = 49,226**	***n* = 42,821**	***n* = 6,405**	
**Demographics**
Age, years	74.8 ± 6.9	74.8 ± 7.0	75.1 ± 6.7	<0.001
Age, *n* (%)				<0.001
65 to <75 years	25,189 (51.2)	22,098 (51.6)	3,091 (48.3)	
75 to <85 years	19,185 (39.0)	16,458 (38.4)	2,727 (42.6)	
85 years or more	4,852 (9.9)	4,265 (10.0)	587 (9.2)	
Male	24,865 (50.5)	21,620 (50.5)	3,245 (50.7)	0.805
**Hospitalized events**
Hospital days	8 [5, 14]	9 [6, 14]	4 [3, 7]	<0.001
>14 hospital days	10,806 (22.0)	10,439 (24.4)	367 (5.7)	<0.001
Death in hospital	1,496 (3.0)	1451 (3.4)	45 (0.7)	<0.001
**Hospitalized costs**
Total costs, $	2,450 [1,390, 7,290]	2,840 [1,520, 8,300]	1,370 [858, 2,100]	<0.001
Examination costs, $	517 [254, 844]	573 [354, 905]	79.9 [12.7, 112]	<0.001
Treatment costs, $	456 [241, 822]	472 [243, 905]	326 [236, 535]	<0.001
Nursing costs, $	23.5 [10.8, 51.1]	26.2 [12.1, 55.1]	11.5 [5.4, 22.3]	<0.001
Pharmacy costs, $	446 [149, 1,090]	544 [208, 1,230]	57.6 [25.5, 184]	<0.001
Material costs, $	551 [155, 2,380]	522 [145, 3,120]	613 [446, 1,010]	0.003
**eFI, median [IQR]**	–	0.111 [0.067, 0.189]	–	–
**eFI≥0.15**, ***n*** **(%)**	–	14,472 (33.8)	–	–
**eFI**, ***n*** **(%)**
eFI ≤ 0.10	–	18,384 (42.9)	–	
0.10 < eFI ≤ 0.20	–	14,502 (33.9)	–	
0.20 < eFI ≤ 0.30	–	6,169 (14.4)	-	
0.30 < eFI ≤ 0.40	–	2,844 (6.6)	–	
eFI > 0.40	-	922 (2.2)	–	

*Values are showed as mean ± standard deviation, median [IQR], or n (%)*.

*eFI, electronic frailty index; IQR, interquartile range*.

### Correlation Between the eFI and CGA-FI

**Figure 3:**
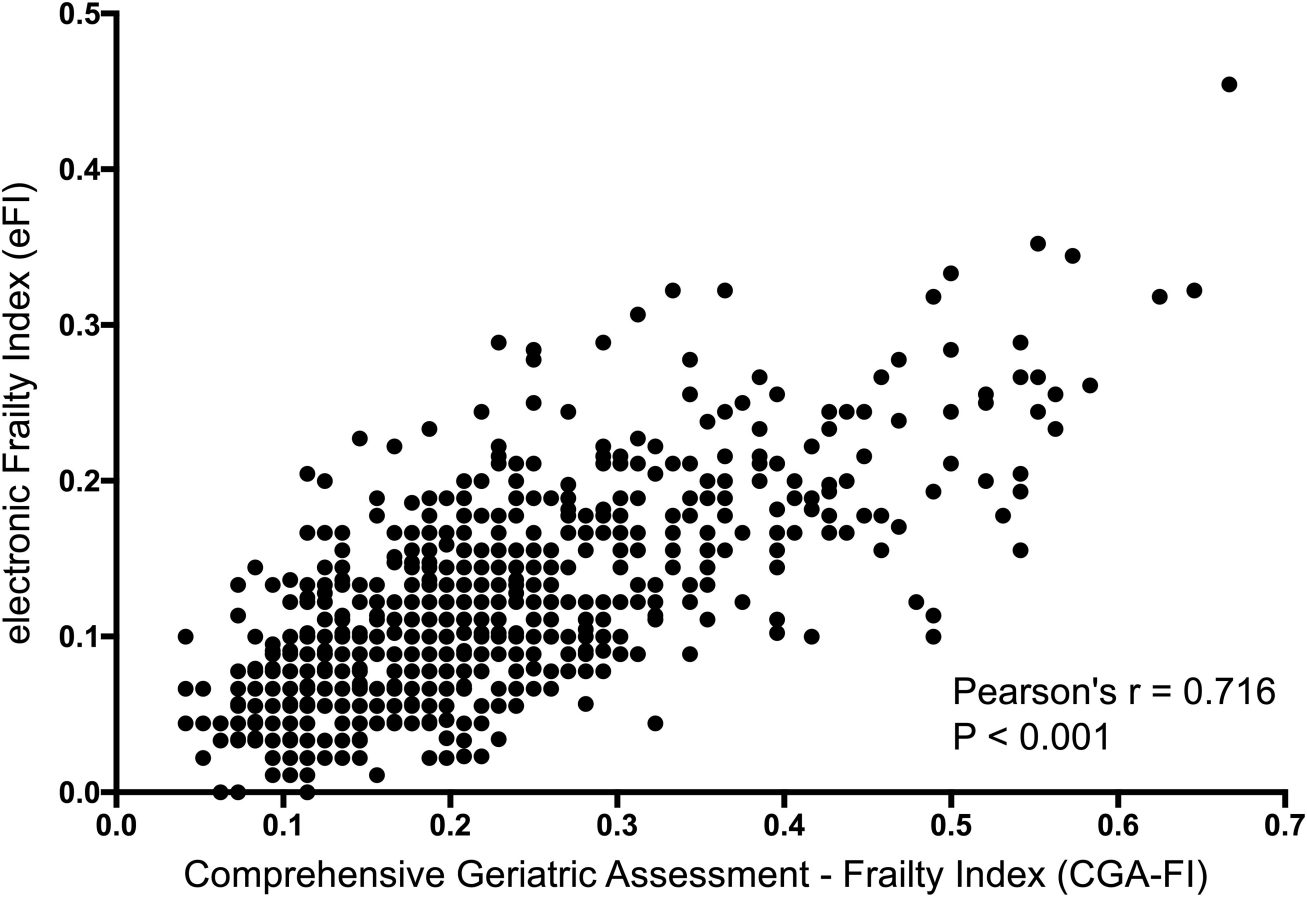
Correlation between the eFI and CGA-FI. eFI, electronic frailty index; CGA-FI, comprehensive geriatric assessment-frailty index.

**Figure 4:**
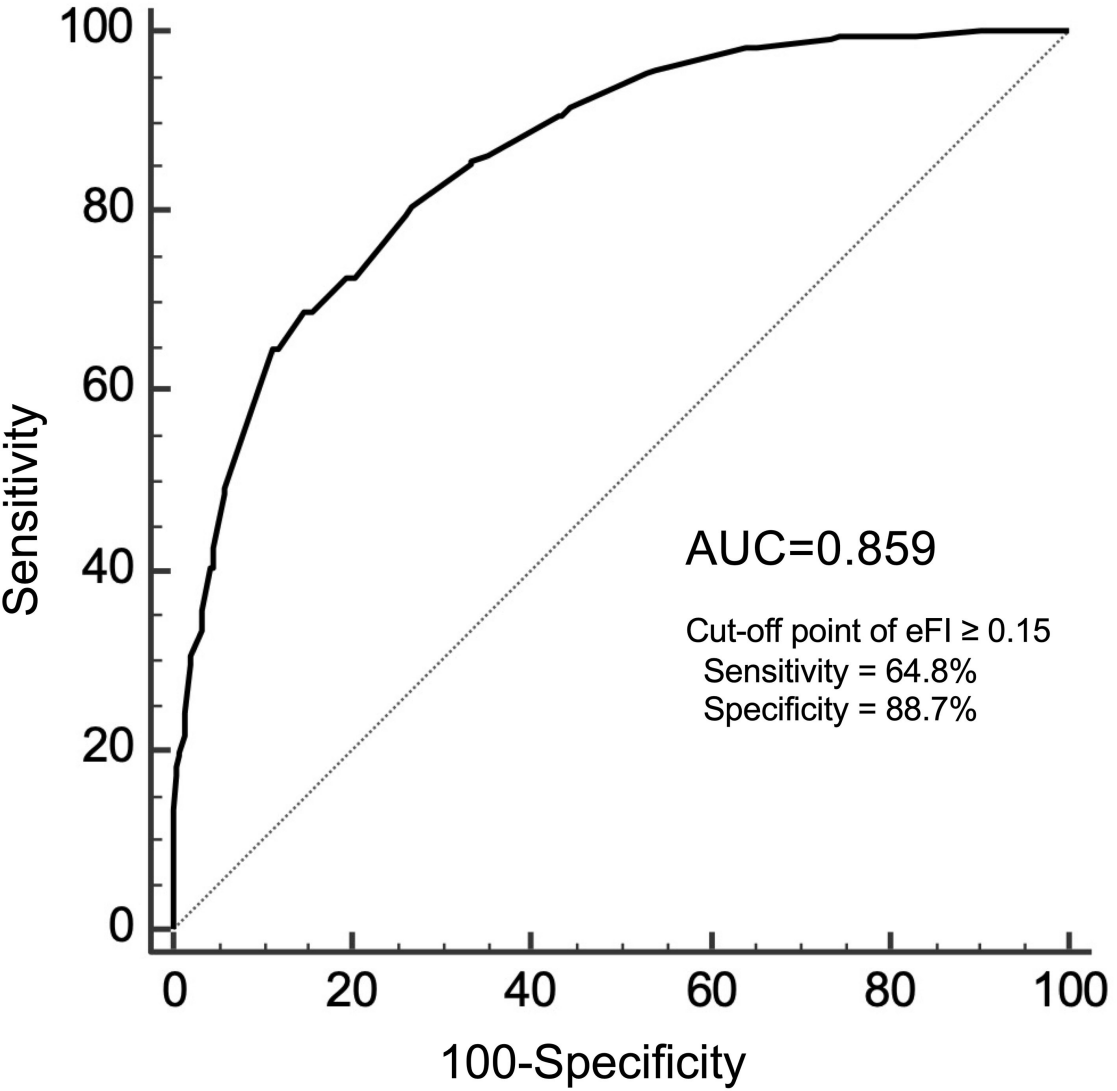
The ROC analysis for the eFI against CGA-FI ≥0.25 to identify frailty. ROC, receiver operating characteristic; eFI, electronic frailty index; CGA-FI, comprehensive geriatric assessment-frailty index; AUC, area under the curve.

In the validation cohort, there were 685 participants who had both the eFI and CGA-FI assessed. The average age was 74.6 ± 6.7 years old, and 48.0% were males. The prevalence of frailty, defined as a CGA-FI ≥ 0.25, was 31.5%. The median CGA-FI was 0.198, while the median eFI was 0.102. Detailed information on all subjects is shown in Supplementary Table 6. A strong correlation is shown between the CGA-FI and eFI (Pearson's *r* = 0.716, *P* < 0.001) (Figure 3). The AUC of the ROC for the eFI to identify frailty in the subjects was 0.859. The sensitivity and specificity to identify frailty were 64.8 and 88.7%, respectively, for an eFI≥0.15 (Figure 4).

### Difference Between the Frail Group and Non-frail Group

We used 0.15 (tertile of eFI) as the cut-off value to indicate frailty in the 42,821 inpatients from the EHR database. Compared with the non-frail group, the frail group was significantly older (78.7 ± 7.1 vs. 72.8 ± 6.0 years; *P* < 0.001) and had a lower percentage of males (46.6 vs. 52.5%; *P* < 0.001). The department distributions and operations were clearly different between these two groups (both *P* < 0.001). The frail group had significantly longer hospital days (12 [8, 18] vs. 8 [5, 13] days, *P* < 0.001) and a higher mortality rate in the hospital (9.3 vs. 0.4%, *P* < 0.001) than the non-frail group. Meanwhile, the total hospitalized cost and each specific cost for the frail group were all significantly higher than those for the non-frail group (all *P* < 0.001) (Table 2).

**Table 2:** Hospitalized events and costs in frail and non-frail groups classified by eFI ≥0.15.

	**Non-frail group**	**Frail group**	***P* values**
	**(eFI <0.15)**	**(eFI≥0.15)**	
	***n* = 28,349**	***n* = 14,472**	
**Demographics**
Age, years	72.8 ± 6.0	78.7 ± 7.1	<0.001
Age, n (%)			<0.001
65 to <75 years	17,939 (63.3)	4,159 (28.7)	
75 to <85 years	9,285 (32.8)	7,173 (49.6)	
85 years or more	1,125 (4.0)	3,140 (21.7)	
Male	14,874 (52.5)	6,746 (46.6)	<0.001
**Departments**			<0.001
Cardiology	5,852 (20.6)	1,689 (11.7)	
Internal (except Cardiology)	8,130 (28.7)	5,553 (38.4)	
Orthopedics	2,101 (7.4)	3,092 (21.4)	
Surgical	11,844 (41.8)	2,102 (14.5)	
(except Orthopedics)			
Emergency and ICUs	422 (1.5)	2,036 (14.1)	
**Operation**			<0.001
Yes	21,528 (75.9)	7,377 (51.0)	
No	6,821 (24.1)	7,095 (49.0)	
**Hospitalized events**			
Hospital days	8 [5, 13]	12 [8, 18]	<0.001
>14 hospital days	4,961 (17.5)	5,478 (37.9)	<0.001
Death in hospital	101 (0.4)	1,350 (9.3)	<0.001
**Hospitalized costs**
Total costs, $	2,290 [1,370, 6,520]	4,790 [2,060, 10,700]	<0.001
Examination costs, $	514 [304, 788]	724 [465, 1,140]	<0.001
Treatment costs, $	429 [217, 766]	588 [303, 1,180]	<0.001
Nursing costs, $	23.2 [10.4, 46.4]	36.2 [16.7, 83.6]	<0.001
Pharmacy costs, $	417 [166, 914]	904 [375, 1,950]	<0.001
Material costs, $	506 [137, 2,590]	568 [160, 5,250]	<0.001

*Values are showed as mean ± standard deviation, median [interquartile range], or n (%)*.

*eFI, electronic frailty index; ICUs, intensive care units*.

### Association of Frailty With Hospitalized Events and Costs

After adjusting for age, sex, and operation, an eFI≥0.15 showed independent correlations with long hospital stay (OR = 2.889, *P* < 0.001) and death in hospital (OR = 19.97, *P* < 0.001) for all older adult inpatients by multivariate logistic regression. In the subgroup analysis for different departments, the eFI was also independently associated with long hospital stay in all subgroups (ORs ranged from 1.392 to 4.750; all *P* < 0.001). The same results are presented for death in hospital (ORs ranged from 7.192 to 34.26; all *P* < 0.001) (Table 3).

**Table 3:** Association between eFI and hospitalized events of elderly inpatients by logistic regression.

**Variables**	**>14 hospital days**				**Death in hospital**			
		**95%CI**				**95%CI**		
	**OR**	**Lower**	**Upper**	***P* values**	**OR**	**Lower**	**Upper**	***P* values**
**All patients (*****n*** **=** **42,821)**	
Age	1.004	1.000	1.007	0.033	1.018	1.011	1.026	<0.001
Sex (female = 1, male = 0)	0.821	0.785	0.860	<0.001	0.624	0.558	0.697	<0.001
Operation (Yes = 1, No = 0)	1.059	1.008	1.113	0.023	0.304	0.269	0.343	<0.001
eFI≥0.15	2.889	2.745	3.040	<0.001	19.97	16.18	24.63	<0.001
**Department of cardiology (*****n*** **=** **7,541)**	
Age	1.008	0.993	1.023	0.274	1.048	1.004	1.093	0.032
Sex (female = 1, male = 0)	0.990	0.823	1.189	0.911	0.832	0.490	1.414	0.497
Operation (Yes = 1, No = 0)	1.116	0.898	1.388	0.323	0.256	0.143	0.458	<0.001
eFI≥0.15	3.342	2.713	4.117	<0.001	7.966	3.903	16.26	<0.001
**Departments of internal medicine (except cardiology) (*****n*** **=** **13,683)**	
Age	1.012	1.006	1.018	<0.001	1.004	0.992	1.015	0.542
Sex (female = 1, male = 0)	0.901	0.836	0.971	0.006	0.679	0.577	0.799	<0.001
Operation (Yes = 1, No = 0)	1.264	1.166	1.370	<0.001	0.703	0.576	0.857	<0.001
eFI≥0.15	2.819	2.598	3.059	<0.001	16.44	12.40	21.78	<0.001
**Department of orthopedics (*****n*** **=** **5,193)**	
Age	1.001	0.993	1.011	0.695	1.092	1.047	1.138	<0.001
Sex (female = 1, male = 0)	0.784	0.691	0.889	<0.001	0.340	0.194	0.595	<0.001
Operation (Yes = 1, No = 0)	2.500	1.934	3.231	<0.001	0.098	0.056	0.169	<0.001
eFI≥0.15	1.392	1.214	1.597	<0.001	7.192	2.168	23.85	0.001
**Surgical Departments (except orthopedics) (*****n*** **=** **13,946)**	
Age	1.001	0.994	1.007	0.864	1.013	0.993	1.034	0.196
Sex (female = 1, male = 0)	0.739	0.680	0.803	<0.001	0.712	0.533	0.952	0.022
Operation (Yes = 1, No = 0)	3.260	2.772	3.834	<0.001	0.409	0.304	0.551	<0.001
eFI≥0.15	3.215	2.886	3.580	<0.001	34.26	22.46	52.26	<0.001
**Emergency department and ICUs (*****n*** **=** **2,458)**	
Age	1.015	1.003	1.028	0.018	1.032	1.016	1.049	<0.001
Sex (female = 1, male = 0)	0.854	0.720	1.012	0.068	0.745	0.601	0.924	0.007
Operation (Yes = 1, No = 0)	0.628	0.518	0.761	<0.001	0.411	0.311	0.542	<0.001
eFI≥0.15	4.750	3.427	6.585	<0.001	22.79	7.224	71.90	<0.001

*eFI, electronic frailty index; CI, confidence interval; OR, odds ratio; ICUs, intensive care units*.

In this study, the hospitalized cost averaged of non-frail and frail group were US $2,290 (1,370, 6,520) and US $4,790 (2,060, 10,700), respectively (Table 1). After adjusting for age, sex, and operation, the eFI was positively associated with total costs (β = 0.453, *P* < 0.001), examination costs (β = 0.269, *P* < 0.001), treatment costs (β = 0.414, *P* < 0.001), nursing costs (β = 0.381, *P* < 0.001), pharmacy costs (β = 0.524, *P* < 0.001), and material costs (β = 0.578, *P* < 0.001) for all older adult inpatients. Subgroups of different departments showed similar correlations between the eFI and hospitalized costs (all *P* < 0.01) (Table 4).

**Table 4:** Association between eFI and hospitalized costs of elderly inpatients by generalized liner regression models.

**Variable**	**Beta-coefficients (95%CI) for eFI (per 0.1)**					
	**Total costs**	**Examination costs**	**Treatment costs**	**Nursing costs**	**Pharmacy costs**	**Material costs**
**All patients (*****n*** **=** **42,821)**					
eFI (per 0.1)	0.453 (0.441–0.465)^**^	0.269 (0.260, 0.278)^**^	0.414 (0.396, 0.432)^**^	0.381 (0.369, 0.394)^**^	0.524 (0.508, 0.540)^**^	0.578 (0.561, 0.594)^**^
**Department of cardiology (*****n*** **=** **7,541)**					
eFI (per 0.1)	0.330 (0.297, 0.364)^**^	0.193 (0.172, 0.213)^**^	0.502 (0.465, 0.539)^**^	0.410 (0.369, 0.450)^**^	0.592 (0.517, 0.667)^**^	0.516 (0.453, 0.578)^**^
**Departments of internal medicine (except cardiology) (*****n*** **=** **13,683)**					
eFI (per 0.1)	0.368 (0.348, 0.388)^**^	0.212 (0.198, 0.225)^**^	0.409 (0.368, 0.450)^**^	0.323 (0.303, 0.344)^**^	0.436 (0.410, 0.462)^**^	0.482 (0.450, 0.514)^**^
**Department of orthopedics (*****n*** **=** **5,193)**					
eFI (per 0.1)	0.072 (0.046, 0.097)^**^	0.193 (0.172, 0.215)^**^	0.108 (0.077, 0.139)^**^	0.165 (0.128, 0.202)^**^	0.287 (0.254, 0.319)^**^	0.047 (0.015, 0.078)^*^
**Surgical departments (except orthopedics) (*****n*** **=** **13,946)**					
eFI (per 0.1)	0.523 (0.497, 0.549)^**^	0.414 (0.391, 0.437)^**^	0.419 (0.378, 0.460)^**^	0.448 (0.420, 0.476)^**^	0.616 (0.582, 0.650)^**^	0.557 (0.521, 0.592)^**^
**Emergency department and ICUs (*****n*** **=** **2,458)**					
eFI (per 0.1)	0.382 (0.349, 0.415)^**^	0.327 (0.299, 0.355)^**^	0.433 (0.392, 0.474)^**^	0.332 (0.293, 0.372)^**^	0.530 (0.481, 0.579)^**^	0.397 (0.353, 0.441)^**^

*The beta coefficients of eFI (per 0.1) on hospitalized costs were calculated by generalized linear regression models with log-linked gamma-distribution, after adjusting for age, sex, and operation*.

*eFI, electronic frailty index; CI, confidence interval; ICUs, intensive care units*.

* *P <0.01 in the generalized liner regression model*.

** *P <0.001 in the generalized liner regression model*.

### Sensitivity Analysis

When using the first hospitalization records instead of the last ones, the mortality in the hospital with sufficient data was 1.4%, which was lower than the 3.4% in the original data. There were 30.0% (12,934/43,116) of patients in the frail group, defined by an eFI≥0.15. All the trends between the frail group and the non-frail group were similar to those from the original analysis (Supplementary Table 7). After the sensitivity analysis, eFI≥0.15 remained an independent risk factor for long hospital stay and death in hospital (Supplementary Table 8). Moreover, the eFI was still independently associated with all kinds of increased hospitalized costs (Supplementary Table 9).

## Discussion

To our knowledge, this is the first study on detecting frailty in older adult inpatients by routine EHR in China. Our study demonstrates that an effective eFI by routine EHR for older adult inpatients can be developed from a general hospital in China, and the eFI was validated by agreement with the CGA-FI. Although the prevalence of frailty varied obviously among different departments, frailty was an independent risk factor for hospitalized events in each subgroup of different departments. As the degree of frailty increased, the hospitalized costs for older adult inpatients increased accordingly.

### Frailty Assessment by Routine EHR

In this study, we constructed an eFI to assess frailty using routine EHR. Our results showed a strong correlation between eFI and CGA-FI, which was consistent with the findings of Brundle et al. in the UK (25) and Abbasi et al. in Canada (31). Previous studies have shown that eFI could predict mid-term adverse events in older adult patients (22, 23). Our study showed that the eFI was independently associated with long hospital stay and death in hospital. Although the eFI opens a new direction to fill the gap between research and clinical application, several details should be considered in clinical practices based on our results.

First, the cut-off value of the eFI for identifying frailty should be redefined according to the specific situation, instead of taking a generally accepted fixed value, such as 0.25 for the CGA-FI. Our study showed that the eFI was lower than the CGA-FI despite both having a strong correlation. The median CGA-FI was 0.198, while the median eFI was only 0.102 in the validation cohort. Therefore, applying cut-off value from CGA-FI to the eFI will underestimate the prevalence of the frail population. A similar study that simultaneously compared their own eFI and CGA-FI also showed that eFI was relatively lower than the CGA-FI for 85 participants in a Canadian primary care program (31). There were several possible reasons why our eFI values were lower than those of the CGA-FI. Our eFI was based on routine EHR from a single hospitalization rather than a summary of medical records across multiple visits. In a single hospitalization, the diagnoses seemingly not highly-related to the current treatments, such as depression or anxiety, may be ignored, especially in surgical departments. Moreover, the nurses might not pay enough attention to the conditions related to frailty during the admission assessment, which might have led to the neglect of some positive manifestations. Given that the frailty prevalence was 35.1% by the CGA-FI in our previous survey for older adult inpatients (21), it was more reasonable to use the tertile of eFI values (0.15) as the cut-off value for identifying frailty. Perhaps each center needs to define its own cut-off value for the eFI according to its own situation and research results.

Second, the timeliness of constructing the eFI should be considered in clinical practice. The routine EHR used to construct the eFI could be mainly collected within 24 h after admission in our study except for the diagnostic codes. Compared with the well-checked, ICD-10 coded discharge diagnosis, the uncoded admission diagnosis was relatively difficult to analyze. Therefore, we used the discharge diagnosis to calculate the eFI. If the admission diagnosis is improved by coding in the future, we would be able to obtain the eFI within 24 h after admission, which would be not only useful in follow-up of frail patients after discharge, but also in guiding to manage older adult inpatients in a targeted manner during hospitalization. In previous studies, the eFI was mainly developed in primary care settings (22, 23, 31). Compared with relatively complete hospitalization records, the cleaning of primary medical data is more complicated. It is difficult to obtain all information from one setting at one visit. There often needs to be 1 or 2 years of medical records to construct an eFI, which leads to a time lag in the guidance of clinical practice.

Third, the eFI should reflect the variability in frail conditions. Frailty is a dynamic condition, and individuals can fluctuate between states of severity of frailty (32). In previous studies, researchers have rarely paid attention to this point when constructing an eFI (22). This might also be due to the limitations of electronic medical data, which caused frailty assessment to rely too much on diagnostic codes (24). Pajewski et al. and Abbasi et al. improved the composition of the eFI by adding some laboratory tests and functional abilities (23, 31). We also agree with this improvement. Therefore, we added a 20-item set of nursing assessments and a 5-item set of laboratory tests to the factors of the eFI. A dynamic eFI during hospitalization can more effectively reflect the current condition of older adult inpatients, which would be more precisely guide clinical interventions.

The primary advantage of EHR data is its large volume, but the biggest challenge of EHR-based studies is the presence of missing data (33). Our study showed that 13.0% of patients had insufficient data to calculate the eFI, while Pajewski's study reported 29.6% (23), others did not report the details. We found that these 13% of patients had a significantly lower proportion of adverse events, which was consistent with the results of Pajewski's study (23). Our study showed that the department of ophthalmology contributed the majority of these patients (54.5%, 3491/6405), while the prevalence of frailty was only 5.07% in the department of ophthalmology. These results indicate that patients with insufficient data had a lower percentage of frailty. Therefore, the eFI can be applied to the vast majority of patients with a need for frailty assessment. Meanwhile, we should consider the tolerance for missing data when we select the factors for constructing an eFI.

### Prevalence of Frailty Among Different Departments

We found that there were significant differences in the prevalence of frailty among the different departments. Generally, emergency departments and ICUs had the highest percentage of frailty (average: 82.8%), followed by internal medicine departments (average: 34.1%), and surgical departments (average: 27.1%). This trend was consistent with the results of a recent multicenter survey on frailty in older adult inpatients in China (34).

In this study, we found that the prevalence of frailty in the department of orthopedics (59.5%) was significantly higher than that in other surgical departments and most internal medicine departments. This was mainly because the conditions for most orthopedic patients were braked by osteoarthritis or fractures, especially hip fractures. The activities of daily living assessments for these inpatients in this state could not reflect their daily conditions. We should keep this point in mind when we use the eFI to guide clinical practice for inpatients in department of orthopedics. In our study, the prevalence of frailty in department of cardiology was lower than that in most internal medicine departments. This result was consistent with results in our previous cohort study (21). Since non-emergency percutaneous coronary intervention is not an outpatient procedure in China, it is easy to be explained. However, we were surprised to find that the prevalence of frailty in the gastroenterology ward (17.7%) was the lowest among all internal medicine departments. This might have been caused by the large amount of endoscopic procedures that lead to the development of gastroenterology near surgical departments. For hospitals that do not overly rely on endoscopic procedures, the prevalence of frailty in the gastroenterology ward may be closer to the average level among internal medical departments. In short, due to the differences in disease characteristics and treatment models, the prevalence of frailty varied significantly among different departments.

### Association Between Frailty and Hospitalized Costs

Our findings showed that the hospitalized cost of patients with frailty was significantly higher than that patients without frailty. The eFI was positively associated with costs after adjusting age, sex and operation. This trend exists in all kinds of hospitalized costs (examination, treatment, nursing, pharmacy and material costs).

Previous studies has also suggested that frailty was associated with healthcare expenditure, both in developed and developing countries (5, 8–11, 15, 16). Several small-sample studies have shown that frailty was associated with increased hospitalized costs for patients with colorectal surgery (6), cardiac surgery (13), or transcatheter aortic value implantation (35). In china, studies also showed similar results in annual healthcare costs (12, 14). However, one study showed that there was no significant difference in hospitalized costs between frail and non-frail groups with elective non-cardiac surgeries (36), which is contrary to our results. These inconsistencies might have been due to the small sample size.

To our knowledge, previous studies did not simultaneously analyze the correlation between hospitalized costs and frailty in different departments. Our results showed that the increases in hospitalized costs in the department of orthopedics were smaller than those in other subgroups, especially in terms of material costs. There are several possible reasons to explain this phenomenon. Firstly, the cost of medical consumable materials is a large part of the total hospitalized cost in the department of orthopedics, whether for frail or non-frail inpatients. Secondly, the cost of medical consumable materials in China depends not only on the patient's condition but also on the patient's own choice. For example, patients with low income or without medical insurance may be more likely to choose the less expensive of similar products. Thirdly, the frailty assessment was influenced by patient braking, which may have led to an overestimation of the degree of frailty in many patients with fracture. However, even so, hospitalized costs are positively related to the degree of frailty for older adult inpatients in the department of orthopedics, after adjusting for age, sex, and operation.

Overall, considering that in-hospital interventions on frailty have been effective for older adult inpatients to reverse the degree of frailty (37), timely recognition of frailty is important to guide interventions on frailty and even reduce hospitalized costs to some degree for older adult inpatients.

## Limitations

There are three main limitations in this study. First, there was no follow-up data after discharge. We could not verify the long-term predictive value of the eFI for adverse events in the older adult inpatients because it is difficult to link our hospital EHR with national resident death records or EHR from other hospitals. Second, we used discharge but not admit diagnoses when building eFI. In our hospital, discharge diagnoses were inspected and coded in a variation of ICD-10 by specialists, which are the best source we can find representing the disease burden of patients at present. One of our targets in the future research is to build eFI based on the information gathered in first 24 h after admission, and to verify the value in prediction of outcomes. Third, despite its relatively large size, the EHR data in this study were from a single general hospital, which may not represent all hospitals in China. Multicenter studies with follow-up data are warranted in the future.

## Conclusion

In summary, we successfully developed an effective eFI using routine EHR for older adult inpatients from a general hospital in China. With good agreement with the CGA-FI, the eFI can be a useful and convenient tool to clinicians for detecting frailty in older adult inpatients. The prevalence of frailty varied obviously among different departments, but frailty was an independent risk factor for long hospital stay and death in hospital. As the degree of frailty increased, the hospitalized costs for older adult inpatients increased accordingly.

## Data Availability Statement

The raw data supporting the conclusions of this article will be made available by the authors, without undue reservation.

## Ethics Statement

The studies involving human participants were reviewed and approved by the Ethics Committee of Beijing Hospital (No. 2019BJYYEC-131-01). The Ethics Committee waived the requirement of written informed consent for participation.

## Author Contributions

Y-DL, JS, J-FY, and HW contributed conception and design of the study. Y-BX performed data collection, preparation, and cleansing. Y-DL and M-HD performed the statistical analysis and wrote the draft of the manuscript. All authors contributed to the article and approved the submitted version.

## Funding

This work was supported by the Non-profit Central Research Institute Fund of Chinese Academy of Medical Sciences, China (No. 2019PT320013); the Beijing Municipal Science and Technology Commission, China (D181100000218003); and the CAMS Innovation Fund for Medical Sciences (No. 2018-I2M-1-002).

## Conflict of Interest

The authors declare that the research was conducted in the absence of any commercial or financial relationships that could be construed as a potential conflict of interest.

## Publisher's Note

All claims expressed in this article are solely those of the authors and do not necessarily represent those of their affiliated organizations, or those of the publisher, the editors and the reviewers. Any product that may be evaluated in this article, or claim that may be made by its manufacturer, is not guaranteed or endorsed by the publisher.
