# Supplementary material for: Development and Validation of an Electronic Frailty Index Using Routine Electronic Health Records: An Observational Study From a General Hospital in China
Source: Front Med (Lausanne). 2021 Sep 28;8:731445. doi: 10.3389/fmed.2021.731445 (PMC8505669; doi:10.3389/fmed.2021.731445)
Supplement: Supplementary file 1 [file Table_1.DOCX]

**eTable 1. Factors of Comprehensive Geriatric Assessment-Frailty Index (CGA-FI)**

| Frailty index | Cut-off |
| --- | --- |
| 1.Need help bathing | Yes=1, No=0 |
| 2. Need help dressing | Yes=1, No=0 |
| 3. Need help using the toilet | Yes=1, No=0 |
| 4. Need help getting in / out of chair | Yes=1, No=0 |
| 5. Need help feeding | Yes=1, No=0 |
| 6. Incontinence | Yes=1, No=0 |
| 7. Need help shopping | Yes=1, No=0 |
| 8. Need help with finances | Yes=1, No=0 |
| 9. Need help using transportation | Yes=1, No=0 |
| 10. Need help using telephone | Yes=1, No=0 |
| 11. Need help managing medications | Yes=1, No=0 |
| 12. Need help with housekeeping | Yes=1, No=0 |
| 13. Need help preparing meals | Yes=1, No=0 |
| 14. Need help washing clothes | Yes=1, No=0 |
| 15. Hypertension | Yes=1, No=0 |
| 16. Coronary heart disease | Yes=1, No=0 |
| 17. Atrial fibrillation | Yes=1, No=0 |
| 18. Congestive heart failure | Yes=1, No=0 |
| 19. Peripheral artery disease | Yes=1, No=0 |
| 20. Chronic Lung disease | Yes=1, No=0 |
| 21. Sleep apnea hypopnea syndrome | Yes=1, No=0 |
| 22. Diabetes | Yes=1, No=0 |
| 23. Thyroid dysfunction | Yes=1, No=0 |
| 24. Peptic ulcer | Yes=1, No=0 |
| 25. Stroke | Yes=1, No=0 |
| 26. Chronic kidney disease | Yes=1, No=0 |
| 27. Osteoarthritis | Yes=1, No=0 |
| 28. Osteoporosis | Yes=1, No=0 |
| 29. Parkinson’s disease | Yes=1, No=0 |
| 30. Cancer | Yes=1, No=0 |
| 31. Depression | Yes=1(GDS-5items≥2), No=0(GDS-5items<2) |
| 32. Anxiety | Yes=1(HADS-A≥8), No=0(HADS-A<8) |
| 33. Loneliness | Yes=1, No=0 |
| 34. Cognition | MMSE<18 =1;  18≤MMSE<24 =0.5;  MMSE≥ 24 =0 |
| 35. Visual impairment | Yes=1, No=0 |
| 36. Hearing impairment | Yes=1, No=0 |
| 37. Chewing impairment | Yes=1, No=0 |
| 38. Fall history | Yes=1, No=0 |
| 39. Chronic constipation | Yes=1, No=0 |
| 40. Chronic pain | Yes=1, No=0 |
| 41. Insomnia | Yes=1(AIS≥6), No=0(AIS<6) |
| 42. Depend on assistive devices | Yes=1, No=0 |
| 43. Take exercise outside | Yes=1, No=0 |
| 44. Body mass index (BMI) (kg/m^2^) | BMI≤18 or BMI≥28 =1;  25≤BMI<28 =0.5;  18<BMI<25 =0 |
| 45. Calf circumference (cm) | ≤25^th^ percentile =1;  25^th^ to 50^th^ percentiles =0.5;  >50^th^ percentile =0 |
| 46. Peak flow (liters/min) |  |
| 47. Grip strength (kg) |  |
| 48. 4m-walking speed (m/s) |  |

Abbreviations: GDS-5items, 5-item Geriatric Depression Scale; HADS-A, Hospital Anxiety and Depression Scale-Anxiety; MMSE, Mini-Mental State Examination; AIS, Athens Insomnia Scale.

**eTable 2. Factors of electronic Frailty Index (eFI) from the routine electronic health records**

| **Factors of eFI** | **Cut-off** |
| --- | --- |
| **Disease from ICD-10 codes (20 items)** | |
| 1.Hypertension | Yes=1, No=0 |
| 2.Heart failure | Yes=1, No=0 |
| 3.Myocardial Infarction | Yes=1, No=0 |
| 4.Atrial fibrillation / Atrial flutter | Yes=1, No=0 |
| 5.Peripheral arterial disease | Yes=1, No=0 |
| 6.Venous thromboembolism | Yes=1, No=0 |
| 7.Chronic lung disease | Yes=1, No=0 |
| 8.Peptic ulcer | Yes=1, No=0 |
| 9.Chronic kidney disease | Yes=1, No=0 |
| 10.Diabetes | Yes=1, No=0 |
| 11.Thyroid dysfunction | Yes=1, No=0 |
| 12.Stroke | Yes=1, No=0 |
| 13.Parkinson’s Disease / Parkinsonism | Yes=1, No=0 |
| 14.Dementia | Yes=1, No=0 |
| 15.Anxiety | Yes=1, No=0 |
| 16.Depression | Yes=1, No=0 |
| 17.Osteoporosis | Yes=1, No=0 |
| 18.Arthritis | Yes=1, No=0 |
| 19.Spondylosis / disc disorders | Yes=1, No=0 |
| 20.Malignancy | Yes=1, No=0 |
| **Nursing assessment from nursing records (20 items)** | |
| 21.Feeding | These 10 items are from Barthel Index. The methods of assessment are according to the guidance of Barthel Index, in which 100 score indicates independence in all activities, while, 0 score indicates dependence in all activities.  The score in eFI = 10 - total score of Barthel Index/10 |
| 22.Bathing |  |
| 23.Grooming |  |
| 24.Dressing |  |
| 25.Bowels |  |
| 26.Bladder |  |
| 27.Toilet use |  |
| 28.transfers (bed to chair and back) |  |
| 29.mobility (on level surfaces) |  |
| 30. stairs |  |
| 31. Visual impairment | Yes=1, No=0 |
| 32. Hearing impairment | Yes=1, No=0 |
| 33. Insomnia | Need drug=1, No need drug=0 |
| 34. Consciousness statement | coma or narcolepsy=1, awake=0 |
| 35. Constipation | Yes=1, No=0 |
| 36. Appetite | bad=1, acceptable or unable to assess=0.5, good=0 |
| 37. Pressure ulcer | Yes=1, No=0 |
| 38. Body mass index (BMI) (kg/m^2^) | BMI≤18 or BMI≥28 =1  25≤BMI<28 =0.5  18<BMI<25 =0 |
| 39.Heart rate (HR) (bpm) | HR<50 or HR >100 =1  50≤HR≤100 =0 |
| 40.Blood pressure (BP) (mmHg)  Systolic BP (SBP)  Diastolic BP (DBP) | SBP<90 or DBP <60 =1  90≤SBP<140 and 60≤ DBP <90 = 0  Else = 0.5 |
| **Laboratory items from hospitalized records (5 items)** | |
| 41.Haemoglobin (HGB) (g/L) | HGB<90 =1  90≤HGB<110 =0.5  HGB≥110 =0 |
| 42.Albumin (ALB) (g/L) | ALB<30 =1  30≤ALB<40 =0.5  ALB≥40 =0 |
| 43.D-dimer (ng/ml DDU) | D-dimer ≥255 =1  D-dimer<255 =0 |
| 44.Sodium (Na) (mmol/L) | Na<125 or ≥150 =1  125≤Na<135 =0.5  135≤Na<150 =0 |
| 45.Urine protein | Positive =1  Negative or trace =0 |

**Notes:** missing values of Lab ≤2 items & missing values of Nursing (except Barthel index) ≤4 items. eFI= sum of items without missing values / number of items without missing values

**eTable 3. ICD-10 Diagnosis code included in the electronic Frailty Index**

| No. | System | Disease | ICD-10 Definition |
| --- | --- | --- | --- |
| 1 | Cardiology | Hypertension | I10-I15 |
| 2 | Cardiology | Heart failure | I50 |
| 3 | Cardiology | Myocardial Infarction | I21-I23, I24.1, I25.2 |
| 4 | Cardiology | Atrial fibrillation / Atrial flutter | I48 |
| 5 | Peripheral vascular disease | Peripheral arterial disease | I73, I74, E10.5, E11.5, E14.5 |
| 6 | Peripheral vascular disease | Venous thromboembolism | I80-I82 |
| 7 | Respiratory system | Chronic lung disease | J40-J47, J84.1, J84.8, J84.9 |
| 8 | Gastroenterology | Peptic ulcer | K25-K28 |
| 9 | Urological system | Chronic kidney disease | N18, N19, E10.2, E11.2, E13.2, I12, I13 |
| 10 | Endocrinology | Diabetes | E10-E14 |
| 11 | Endocrinology | Thyroid dysfunction | E01, E03, E05, E06 |
| 12 | Neurology | Stroke | I60, I61, I62.9, I63, I64, I69.0, I69.1, I69.2, I69.3, I69.4 |
| 13 | Neurology | Parkinson Disease / Parkinsonism | G20, G21, G22 |
| 14 | Neurology | Dementia | G30, F00-F03 |
| 15 | Psychiatry | Anxiety | F40, F41, F06.4 |
| 16 | Psychiatry | Depression | F31.3-F31.5, F32, F33, F41.2, F06.3 |
| 17 | Musculoskeletal system | Osteoporosis | M80, M81, S32.0, S22.0 |
| 18 | Musculoskeletal system | Arthritis | M05-M19 |
| 19 | Musculoskeletal system | Spondylosis / disc disorders | M47, M48.0, M50, M51 |
| 20 | Oncology | Malignancy | C00-C97 |

**eTable 4.** Description of missing data elements for routine electronic health records to calculate the electronic Frailty Index (eFI)

|  | All patients  n = 49,226 | Sufficient data to calculate eFI  n = 42,821 | Insufficient data to calculate eFI  n = 6,405 |
| --- | --- | --- | --- |
| No. of Missing Diagnosis items, n (%) | 0 (0.0) | 0 (0.0) | 0 (0.0) |
| No. of Missing Barthel Index, n (%) | 17 (<0.1) | 0 (0.0) | 17 (0.3) |
| No. of Missing Nursing and Lab items, n (%) |  |  |  |
| Visual impairment | 714 (1.5) | 91 (0.2) | 623 (9.7) |
| Hearing impairment | 826 (1.7) | 192 (0.5) | 634 (9.9) |
| Insomnia | 728 (1.5) | 110 (0.3) | 618 (9.7) |
| Consciousness statement | 836 (1.7) | 212 (0.5) | 624 (9.7) |
| Constipation | 643 (1.3) | 24 (0.1) | 619 (9.7) |
| Appetite | 1,228 (2.5) | 570 (1.3) | 658 (10.3) |
| Pressure ulcer | 2,040 (4.1) | 1,382 (3.2) | 658 (10.3) |
| Body mass index | 4,196 (8.5) | 4,064 (9.5) | 132 (2.1) |
| Heart rate | 19 (<0.1) | 0 (0.0) | 19 (<0.1) |
| Blood pressure | 23 (<0.1) | 3 (<0.1) | 20 (0.3) |
| Haemoglobin | 3,023 (6.1) | 181 (0.4) | 2,842 (44.4) |
| Albumin | 7,775 (15.8) | 1,968 (4.6) | 5,807 (90.7) |
| D-dimer | 9,477 (19.3) | 3,943 (9.2) | 5,534 (86.4) |
| Sodium | 6,021 (12.2) | 270 (0.6) | 5,751 (89.8) |
| Urine protein | 6,526 (13.3) | 3,577 (8.4) | 2,949 (46.0) |
| Total number of Missing Nursing and Lab items, n (%) |  |  |  |
| Zero | 30,135 (61.2) | 30,135 (70.4) | 0 (0.0) |
| One | 9,048 (18.4) | 9,048 (21.1) | 0 (0.0) |
| Two | 3,400 (6.9) | 3,400 (7.9) | 0 (0.0) |
| Three | 3,079 (6.3) | 215 (0.5) | 2,864 (44.7) |
| Four | 570 (1.2) | 21 (<0.1) | 549 (8.6) |
| Five | 2,287 (4.6) | 2 (<0.1) | 2,285 (35.7) |
| Six | 86 (0.2) | 0 (0.0) | 86 (1.3) |
| Seven | 460 (0.9) | 0 (0.0) | 460 (7.2) |
| Eight | 83 (0.2) | 0 (0.0) | 83 (1.3) |
| Nine | 29 (0.1) | 0 (0.0) | 29 (0.5) |
| Ten | 5 (<0.1) | 0 (0.0) | 5 (0.1) |
| Eleven | 10 (<0.1) | 0 (0.0) | 10 (0.2) |
| Twelve | 18(<0.1) | 0 (0.0) | 18(0.3) |
| Thirteen | 1 (<0.1) | 0 (0.0) | 1 (<0.1) |
| Fourteen | 0 (0.0) | 0 (0.0) | 0 (0.0) |
| Fifteen | 15 (<0.1) | 0 (0.0) | 15 (0.2) |

**eTable 5. Departments of all patients stratified by whether or not the electronic frailty index (eFI) could be calculated from the routine electronic health records**

|  | All patients  n = 49,226 | Sufficient data to calculate eFI  n = 42,821 | Insufficient data to calculate eFI  n = 6,405 |
| --- | --- | --- | --- |
| **Departments,** n (%) |  |  |  |
| Cardiology | 7,603 (15.4) | 7,541 (17.6) | 62 (1.0) |
| Gastroenterology | 1,948 (4.0) | 1,930 (4.5) | 18 (0.3) |
| Oncology Center | 2,178 (4.4) | 1,972 (4.6) | 206 (3.2) |
| Dermatology | 214 (0.4) | 213 (0.5) | 1 (<0.1) |
| General Medicine | 708 (1.4) | 658 (1.5) | 50 (0.8) |
| Immunology | 537 (1.1) | 529 (1.2) | 8 (0.1) |
| Endocrinology | 1,227 (2.5) | 1,214 (2.8) | 13 (0.2) |
| Traditional Chinese Medicine | 722 (1.5) | 216 (0.5) | 506 (7.9) |
| Respiratory Medicine | 3,354 (6.8) | 3,307 (7.7) | 47 (0.7) |
| Hematology | 452 (0.9) | 449 (1.0) | 3 (<0.1) |
| Neurology | 1,601 (3.3) | 1,555 (3.6) | 46 (0.7) |
| Nephrology | 862 (1.8) | 845 (2.0) | 17 (0.3) |
| Geriatrics | 682 (1.4) | 678 (1.6) | 4 (0.1) |
| Rehabilitation | 123 (0.2) | 117 (0.3) | 6 (0.1) |
| Orthopaedics | 5,254 (10.7) | 5,193 (12.1) | 61 (1.0) |
| Plastic Surgery | 48 (0.1) | 41 (0.1) | 7 (0.1) |
| Otolaryngology | 798 (1.6) | 747 (1.7) | 51 (0.8) |
| Ophthalmology | 5,128 (10.4) | 1,637 (3.8) | 3,491 (54.5) |
| Urology | 2,875 (5.8) | 2,837 (6.6) | 38 (0.6) |
| Gynecology | 1,192 (2.4) | 837 (2.0) | 355 (5.5) |
| Thoracic Surgery | 1,220 (2.5) | 1,196 (2.8) | 24 (0.4) |
| Stomatology | 162 (0.3) | 148 (0.3) | 14 (0.2) |
| General Surgery | 5,505 (11.2) | 4,221 (9.9) | 1,284 (20.0) |
| Cardiac Surgery | 305 (0.6) | 297 (0.7) | 8 (0.1) |
| Vascular Surgery | 846 (1.7) | 828 (1.9) | 18 (0.3) |
| Neurosurgery | 1,182 (2.4) | 1,157 (2.7) | 25 (0.4) |
| Surgical ICU | 97 (0.2) | 97 (0.2) | 0 (0.0) |
| Respiratory ICU | 280 (0.6) | 278 (0.6) | 2 (<0.1) |
| Cardiac ICU | 913 (1.9) | 895 (2.1) | 18 (0.3) |
| Emergency Medicine | 1,210 (2.5) | 1,188 (2.8) | 22 (0.3) |

Abbreviations: eFI, electronic frailty index; ICU, intensive care unit.

**eTable 6. Characteristics of patients in cohort study**

|  | patients in cohort study with both CGA-FI and eFI  n = 685 |
| --- | --- |
| **Demographics** |  |
| Age, years | 74.6 ± 6.7 |
| Age, No. (%) |  |
| 65 to <75 years | 357 (52.1) |
| 75 to <85 years | 268 (39.1) |
| 85 years or more | 60 (8.8) |
| Male | 329 (48.0) |
| **Hospitalized events** |  |
| Hospital days | 7 [5, 12] |
| >14 hospital days | 115 (16.8) |
| Death in hospital | 4 (0.6) |
| **CGA-FI,** median [IQR] | 0.198 [0.135, 0.271] |
| **CGA-FI ≥0.25**, n (%) | 216 (31.5) |
| **eFI,** median [IQR] | 0.102 [0.070, 0.156] |
| **eFI≥0.15,** n (%) | 193 (28.2) |
| **eFI,** n (%) |  |
| eFI ≤ 0.10 | 286 (41.8) |
| 0.10 < eFI ≤0.20 | 312 (45.5) |
| 0.20 < eFI ≤0.30 | 77 (11.2) |
| 0.30 < eFI ≤0.40 | 9 (1.3) |
| eFI > 0.40 | 1 (0.1) |

Notes: Values are showed as mean ± standard deviation, median [IQR], or n (%). Abbreviations: CGA-FI, comprehensive geriatric assessment-frailty index; eFI, electronic frailty index; IQR, interquartile range.

**eTable 7. Sensitivity Analysis: Hospitalized events and costs in frail and non-frail groups classified by eFI ≥0.15**

|  | Sufficient data to calculate eFI  n = 43,116 | Non-frail group  (eFI<0.15)  n = 30,182 | Frail group  (eFI≥0.15)  n = 12,934 | P values |
| --- | --- | --- | --- | --- |
| **Demographics** |  |  |  |  |
| Age, years | 74.2 ± 6.8 | 72.5 ± 6.0 | 78.0 ± 7.1 | <0.001 |
| Age, n (%) |  |  |  | <0.001 |
| 65 to <75 years | 23,460 (54.4) | 19,402 (64.3) | 4,058 (31.4) |  |
| 75 to <85 years | 16,148 (37.5) | 9,733 (32.2) | 6,415 (49.6) |  |
| 85 years or more | 3,508 (8.1) | 1,047 (3.5) | 2,461 (19.0) |  |
| Male | 21,747 (50.4) | 15,885 (52.6) | 5,862 (45.3) | <0.001 |
| **Departments** |  |  |  | <0.001 |
| Cardiology | 7,837 (18.2) | 6,370 (21.1) | 1,467 (11.3) |  |
| Internal (except Cardiology) | 13,273 (30.8) | 8,500 (28.2) | 4,773 (36.9) |  |
| Orthopaedics | 5,277 (12.2) | 2,187 (7.2) | 3,090 (23.9) |  |
| Surgical (except Orthopaedics) | 14,486 (33.6) | 12,627 (41.8) | 1,859 (14.4) |  |
| Emergency and ICUs | 2,243 (5.2) | 498 (1.6) | 1,745 (13.5) |  |
| **Operation** |  |  |  | <0.001 |
| Yes | 30,247 (70.2) | 23,054 (76.4) | 7,193 (55.6) |  |
| No | 12,869 (29.8) | 7,128 (23.6) | 5,741 (44.4) |  |
| **Hospitalized events** |  |  |  |  |
| Hospital days | 10 [6, 15] | 8 [6, 13] | 13 [8, 18] | <0.001 |
| >14 hospital days | 11,071 (25.7) | 6,130 (20.3) | 4,941 (38.2) | <0.001 |
| Death in hospital | 588 (1.4) | 47 (0.2) | 541 (4.2) | <0.001 |
| **Hospitalized costs** |  |  |  |  |
| Total costs, $ | 2,890 [1,540, 8,460] | 2,410 [1,410, 7,040] | 4,830 [2,100, 10,600] | <0.001 |
| Examination costs, $ | 599 [381, 933] | 550 [341, 845] | 732 [479, 1,120] | <0.001 |
| Treatment costs, $ | 452 [224, 849] | 418 [205, 760] | 543 [270, 1,050] | <0.001 |
| Nursing costs, $ | 20.6 [10.7, 46.4] | 18.4 [9.0, 39.6] | 26.5 [14.4, 62.5] | <0.001 |
| Pharmacy costs, $ | 562 [222, 1,280] | 448 [181, 1,020] | 933 [400, 1,850] | <0.001 |
| Material costs, $ | 526 [156, 3,650] | 512 [151, 2,750] | 570 [166, 6,060] | <0.001 |

Notes: Values are showed as mean ± standard deviation, median [interquartile range], or n (%). Abbreviations: eFI, electronic frailty index; ICUs, intensive care units.

**eTable 8. Sensitivity Analysis: Associations between eFI and hospitalized events of elderly inpatients by logistic regression**

|  | **>14 hospital days** | | | |  | **Death in hospital** | | | |
| --- | --- | --- | --- | --- | --- | --- | --- | --- | --- |
| Variables |  | 95%CI | |  |  |  | 95%CI | |  |
|  | OR | Lower | Upper | P values |  | OR | Lower | Upper | P values |
| All patients (n=43,116) | | | | | | | | | |
| Age | 1.002 | 0.998 | 1.005 | 0.374 |  | 1.043 | 1.031 | 1.055 | <0.001 |
| Gender (female=1, male=0) | 0.824 | 0.789 | 0.862 | <0.001 |  | 0.578 | 0.487 | 0.685 | <0.001 |
| Operation (Yes=1, No=0) | 1.173 | 1.117 | 1.233 | <0.001 |  | 0.334 | 0.278 | 0.400 | <0.001 |
| eFI≥0.15 | 2.527 | 2.404 | 2.657 | <0.001 |  | 18.12 | 13.30 | 24.67 | <0.001 |
| Department of Cardiology (n=7,837) | | | | | | | | | |
| Age | 1.018 | 1.004 | 1.033 | 0.011 |  | 1.057 | 0.928 | 1.125 | 0.083 |
| Gender (female=1, male=0) | 0.744 | 0.626 | 0.885 | <0.001 |  | 0.691 | 0.316 | 1.510 | 0.354 |
| Operation (Yes=1, No=0) | 1.301 | 1.045 | 1.619 | 0.018 |  | 0.267 | 0.115 | 0.620 | 0.002 |
| eFI≥0.15 | 2.854 | 2.340 | 3.480 | <0.001 |  | 8.006 | 2.983 | 21.49 | <0.001 |
| Departments of Internal Medicine (except Cardiology) (n=13,273) | | | | | | | | | |
| Age | 1.009 | 1.004 | 1.015 | 0.002 |  | 1.041 | 1.020 | 1.062 | <0.001 |
| Gender (female=1, male=0) | 0.898 | 0.833 | 0.967 | 0.004 |  | 0.649 | 0.488 | 0.862 | 0.003 |
| Operation (Yes=1, No=0) | 1.444 | 1.335 | 1.561 | <0.001 |  | 0.756 | 0.538 | 1.062 | 0.107 |
| eFI≥0.15 | 2.481 | 2.287 | 2.691 | <0.001 |  | 15.37 | 9.349 | 25.28 | <0.001 |
| Department of Orthopaedics (n=5,277) | | | | | | | | | |
| Age | 1.005 | 0.996 | 1.013 | 0.316 |  | 1.088 | 1.035 | 1.143 | <0.001 |
| Gender (female=1, male=0) | 0.849 | 0.750 | 0.961 | <0.001 |  | 0.324 | 0.163 | 0.645 | 0.001 |
| Operation (Yes=1, No=0) | 3.317 | 2.453 | 4.485 | <0.001 |  | 0.070 | 0.036 | 0.137 | <0.001 |
| eFI≥0.15 | 1.319 | 1.154 | 1.508 | <0.001 |  | 5.768 | 1.684 | 19.76 | 0.005 |
| Surgical Departments (except Orthopaedics) (n=14,486) | | | | | | | | | |
| Age | 1.001 | 0.995 | 1.007 | 0.748 |  | 1.033 | 1.005 | 1.063 | 0.022 |
| Gender (female=1, male=0) | 0.746 | 0.690 | 0.806 | <0.001 |  | 0.566 | 0.373 | 0.860 | 0.008 |
| Operation (Yes=1, No=0) | 4.294 | 3.612 | 5.105 | <0.001 |  | 0.509 | 0.330 | 0.786 | 0.002 |
| eFI≥0.15 | 2.811 | 2.519 | 3.137 | <0.001 |  | 26.28 | 15.40 | 44.84 | <0.001 |
| Emergency Department and ICUs (n=2,243) | | | | | | | | | |
| Age | 1.009 | 0.995 | 1.022 | 0.210 |  | 1.029 | 1.007 | 1.052 | 0.009 |
| Gender (female=1, male=0) | 0.916 | 0.765 | 1.097 | 0.341 |  | 0.746 | 0.557 | 1.000 | 0.050 |
| Operation (Yes=1, No=0) | 0.604 | 0.496 | 0.736 | <0.001 |  | 0.353 | 0.241 | 0.517 | <0.001 |
| eFI≥0.15 | 3.358 | 2.529 | 4.460 | <0.001 |  | 19.86 | 4.863 | 81.10 | <0.001 |

Abbreviations: eFI, electronic frailty index; CI, confidence interval; OR, odds ratio; ICUs, intensive care units.

**eTable 9. Sensitivity Analysis: Associations between eFI and hospitalized costs of elderly inpatients by generalized liner regression models**

|  | **Beta-coefficients (95%CI) for eFI (per 0.1)** | | | | | |
| --- | --- | --- | --- | --- | --- | --- |
| **Variable** | Total | Examination | Treatment | Nursing | Pharmacy | Material |
| All patients (n=43,116) | | | | |  |  |
| eFI (per 0.1) | 0.424 (0.412-0.435) ** | 0.233 (0.224, 0.242) ** | 0.377 (0.359, 0.395) ** | 0.351 (0.338, 0.364) ** | 0.456 (0.440, 0.472) ** | 0.539 (0.522, 0.556) ** |
| Department of Cardiology (n=7,837) | | | | |  |  |
| eFI (per 0.1) | 0.284 (0.249, 0.318) ** | 0.180 (0.160, 0.199) ** | 0.425 (0.386, 0.463) ** | 0.358 (0.313, 0.403) ** | 0.513 (0.431, 0.595) ** | 0.458 (0.395, 0.521) ** |
| Departments of Internal Medicine (except Cardiology) (n=13,273) | | | | |  |  |
| eFI (per 0.1) | 0.310 (0.289, 0.330) ** | 0.186 (0.172, 0.200) ** | 0.359 (0.315, 0.403) ** | 0.293 (0.270, 0.315) ** | 0.351 (0.326, 0.376) ** | 0.400 (0.364, 0.436) ** |
| Department of Orthopedics (n=5,277) | | | | |  |  |
| eFI (per 0.1) | 0.073 (0.049, 0.098) ** | 0.191 (0.171, 0.211) ** | 0.109 (0.080, 0.138) ** | 0.207 (0.170, 0.244) ** | 0.276 (0.247, 0.304) ** | 0.053 (0.023, 0.083) ** |
| Surgical Departments (except Orthopedics) (n=14,486) | | | | |  |  |
| eFI (per 0.1) | 0.534 (0.508, 0.560) ** | 0.410 (0.388, 0.433) ** | 0.428 (0.392, 0.463) ** | 0.447 (0.418, 0.476) ** | 0.609 (0.575, 0.643) ** | 0.578 (0.542, 0.614) ** |
| Emergency Department and ICUs (n=2,243) | | | | |  |  |
| eFI (per 0.1) | 0.318 (0.285, 0.351) ** | 0.270 (0.243, 0.298) ** | 0.386 (0.342, 0.430) ** | 0.281 (0.238, 0.324) ** | 0.473 (0.421, 0.525) ** | 0.320 (0.275, 0.365) ** |

Notes: The beta coefficients of eFI (per 0.1) on hospitalized costs were calculated by generalized linear regression models with log-linked gamma-distribution, after adjusting for age, gender, and operation.

Abbreviations: eFI, electronic frailty index; CI, confidence interval; ICUs, intensive care units.

**: P<0.001 in the generalized liner regression model.
